# Gestational surrogacy at a moral crossroads: autonomy, exploitation, and abolitionism

**DOI:** 10.3389/fgwh.2026.1973804

**Published:** 2026-10-29

**Authors:** Yaakov Bentov, Joseph Schenker

**Affiliations:** 1 Faculty of Medicine, Hebrew University, Jerusalem, Israel; 2 Departments Obstetrics Gynaecology, Hadassah Medical Centre, Jerusalem, Israel

**Keywords:** abolitionism, altruistic, casablanca declaration, commercial, gestational carrier, surrogacy

## Abstract

Gestational surrogacy has become an increasingly important pathway to parenthood for individuals and couples who cannot otherwise achieve or safely sustain pregnancy, yet its expansion has intensified longstanding ethical and regulatory controversy. Drawing on an extensive review of the literature summarized in Supplementary Table 1, this narrative review synthesizes contemporary ethical arguments and regulatory debates concerning gestational surrogacy. The analysis focuses on the tension between reproductive autonomy and concerns regarding exploitation, commodification, structural inequality, and the welfare of children. This synthesis identifies a growing challenge to the traditional binary distinction between commercial and altruistic surrogacy: payment alone does not determine whether an arrangement is exploitative, while altruistic models may also involve coercive pressures arising from familial obligations, social expectations, or economic dependence. At the same time, the child's interests, including secure legal parentage, citizenship, knowledge of origins, and recognition of the gestational relationship, have moved closer to the center of ethical analysis. Regulatory approaches remain highly heterogeneous, ranging from permissive commercial systems to altruistic-only models and complete prohibition. Against a broadly regulationist academic and professional literature, recent political initiatives, most notably the 2023 Casablanca Declaration and increasingly critical European parliamentary discourse, have strengthened an abolitionist framing of surrogacy as a threat to human dignity and a potential form of reproductive exploitation. We argue that the contemporary debate has therefore reached a moral crossroads: the central question is no longer simply whether surrogacy should be commercial or altruistic, but whether exploitation is intrinsic to gestational surrogacy or contingent on the conditions under which it is practiced. A coherent future framework should protect gestational carriers and children while respecting reproductive autonomy, and should address the transnational nature of surrogacy through stronger regulatory coordination.

## 1 Introduction

The introduction of *in vitro* fertilization (IVF) in the late 1970s transformed infertility treatment and enabled new forms of family building, including gestational surrogacy in its contemporary form. Although gestational surrogacy avoids the genetic and gestational dual role characteristic of traditional surrogacy, it has remained ethically controversial since its inception (1). At its core lies a persistent moral tension: gestational surrogacy may allow individuals and couples who cannot otherwise carry a pregnancy to establish a family, yet it also requires another person to undertake the physical and psychological burdens of pregnancy with the prior intention of relinquishing parental responsibility after birth. The scale of the practice is difficult to establish because no international registry systematically records births following surrogacy. The most recent international review cites an earlier estimate of approximately 5,000–15,000 births annually while emphasizing that reliable contemporary global figures are unavailable (2). In the United States, 4.4% of ART births involved gestational carriers in 2021, underscoring the growing clinical relevance of the practice (3).

**Figure 1:**
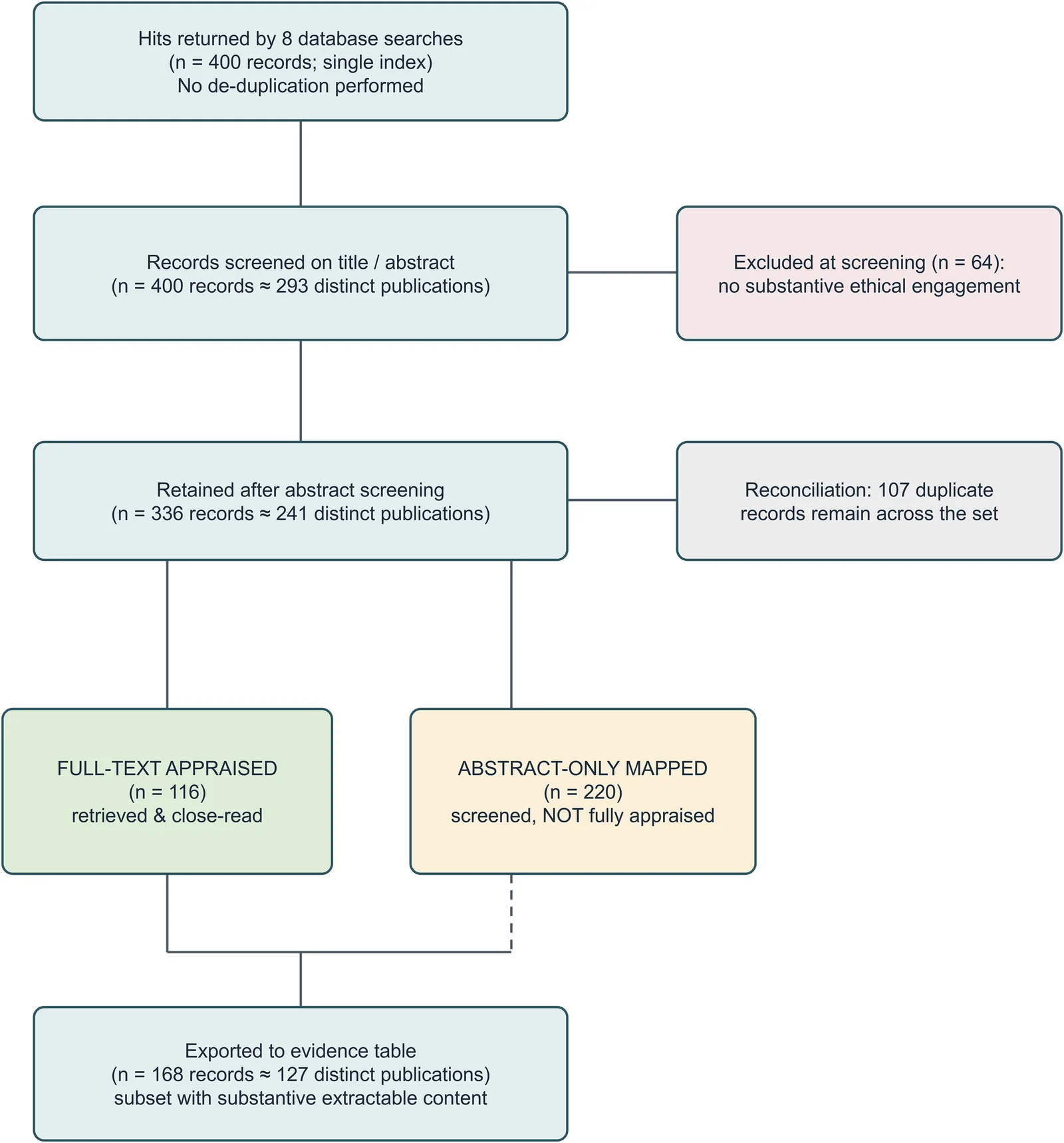
describes the process of the database creation.

A gestational carrier is a person who becomes pregnant, carries, and delivers a child on behalf of intended parent(s), who assume parental responsibility after birth. Surrogacy encompasses arrangements in which the carrier has no genetic relationship to the child, as well as traditional surrogacy in which the carrier provides the oocyte. In gestational surrogacy, embryos may be created using the gametes of both intended parents, donor gametes, donated embryos, or a combination of an intended parent's and a donor's gametes. Arrangements are also commonly classified as altruistic or commercial. In altruistic surrogacy, compensation is generally limited to pregnancy-related expenses, whereas commercial arrangements provide payment beyond reimbursement and are frequently mediated by agencies. These distinctions have major ethical and legal consequences, although, as discussed below, their moral significance has increasingly been challenged.

Despite its growing use, surrogacy remains the subject of marked legal, ethical, and social disagreement (4–6). European regulation is particularly heterogeneous: a review of medically assisted reproduction legislation across 44 countries found surrogacy permitted in 15, with substantial differences in eligibility, compensation, and recognition of parentage (7). These differences reflect competing views of reproductive autonomy, the risk of exploitation, and the welfare of children (8, 9). The European Court of Human Rights has emphasized protection of the best interests and legal identity of children born through surrogacy, but national approaches remain inconsistent (10, 11). Professional organizations have generally favored carefully regulated, non-commercial gestational surrogacy. ESHRE and FIGO emphasize informed consent, protection of gestational carriers, and child welfare, while ACOG similarly stresses medical, psychological, and legal safeguards [ACOG (12)]. This review examines how the moral and regulatory debate has evolved, focusing on indications and access, compensation, competing rights and interests, the commercial-altruistic distinction, the rise of abolitionist arguments, and the prospects for international regulatory coordination.

## 2 Methodology

The following methodology was used to create the database: Literature was identified through eight parallel keyword searches of a single academic index (Elicit's paper search, drawing primarily on the Semantic Scholar corpus), each restricted to publications from 2015 onward (filter year > 2014, no upper bound) and capped at 50 records per query, with no language restriction. The eight searches returned 400 records, which were concatenated without a de-duplication step; deterministic matching on DOI, Semantic Scholar identifier, and normalised title subsequently established that these 400 records corresponded to 293 distinct publications. Titles and abstracts were screened for substantive engagement with the ethics or morality of gestational surrogacy by a single automated relevance classifier applying one inclusion instruction at a confidence threshold of 55 of 100; this excluded 64 records and retained 336 (241 distinct publications). No independent second screener was used, and inter-rater reliability was therefore not assessed. Full text was sought for all 336 retained records by automated best-effort retrieval and was obtained for 116, while 220 remained available at abstract level only. Structured data (central thesis, position on commercial versus altruistic surrogacy, handling of competing interests, cultural/national context, and normative stance) were then extracted by a single automated language-model pass with a fixed quote-supported schema; records for which extraction returned substantive content on at least one dimension were exported to the evidence table (168 records, 127 distinct publications), with the remaining records omitted rather than recorded as empty. Non-English sources (Spanish, French, Italian, Ukrainian, and Lithuanian) were screened and extracted in their original language. The full-text-retrieved records supported close reading and appraisal, whereas the abstract-only records were mapped at the abstract level only and were not treated as fully appraised studies; because no de-duplication or dual-reviewer adjudication was applied, the corpus constitutes a PRISMA-style abstract-level map of the field rather than a registered systematic review. See a description of the database creation process in Figure 1.

A document describing this process in more detail was added as a supplementary file. The choice of which papers to include in the actual review and expend upon was all done manually to generate and support the main claims of the paper.

## 3 Types of surrogacy

Surrogacy is conventionally divided into traditional and gestational forms. In traditional surrogacy, the carrier also provides the oocyte and is therefore genetically related to the child; conception may occur through intercourse or artificial insemination. Although historically important, this model is now uncommon in contemporary reproductive medicine and presents distinctive questions concerning parentage because the person who carries the pregnancy is also the genetic mother. In gestational surrogacy, by contrast, an embryo created through IVF from the gametes of intended parents and/or donors is transferred to a carrier who has no genetic relationship to the resulting child. Gestational surrogacy is now the predominant clinical model. Nevertheless, absence of a genetic relationship does not by itself resolve legal parentage: in several jurisdictions, the person who gives birth is initially recognized as the legal mother, requiring a court order, adoption, or another formal mechanism to establish the intended parents' legal status (1).

## 4 Indications for surrogacy

Gestational surrogacy was initially developed primarily for individuals unable to carry a pregnancy because of medical or anatomical factors. The clearest indication is absolute uterine-factor infertility, including congenital absence of the uterus such as Mayer-Rokitansky-Küster-Hauser syndrome, which occurs in approximately 1 in 4,000–5,000 female newborns (13), or acquired loss of uterine function following obstetric hysterectomy, malignancy, severe intrauterine adhesions, uterine malformations, or unsuccessful reconstructive surgery. Surrogacy may also be considered when pregnancy would impose an unacceptable medical risk, including selected cases of severe cardiovascular disease, advanced renal disease, severe hypertension, uncontrolled diabetes, or autoimmune disease (14). Less clearly defined indications include an unfavorable uterine environment associated with repeated implantation failure or recurrent pregnancy loss.

The ethical landscape becomes more complex when surrogacy is requested for non-medical or social indications. In some jurisdictions it is available to male same-sex couples, single men, and women who do not wish to undergo pregnancy for personal, psychological, or professional reasons (15, 16). Expansion beyond conventional medical indications has shifted part of the debate from treatment of infertility toward broader questions of reproductive liberty, equality of access, and whether the ability to commission gestation should be understood as an extension of reproductive autonomy or as a practice that requires independent ethical justification.

## 5 Moral challenges presented by surrogacy

Gestational surrogacy creates ethical challenges that extend beyond the conventional risks of assisted reproduction. Clinicians may encounter conflicts between the wishes of intended parents and those of the gestational carrier, particularly when decisions concern prenatal testing, fetal reduction, termination of pregnancy, mode or timing of delivery, or maternal risk. Ethical guidance consistently recognizes that the gestational carrier retains bodily autonomy and must remain the principal decision-maker regarding medical interventions during pregnancy (12).

Contemporary gestational surrogacy involves IVF using an oocyte provided by the intended mother or a donor rather than the gestational carrier, and commonly uses frozen–thawed embryos, which accounted for 90.1% of gestational-carrier transfer cycles in a large United States cohort from 2014 to 2020 (17). Medical risk is ethically relevant because gestational carriers undertake pregnancy to enable others to become parents, but adverse outcomes observed in these pregnancies should not automatically be attributed to surrogacy itself. In a retrospective within-woman comparison of singleton births, Woo et al. (18) reported higher rates of preterm birth, low birth weight, and several obstetric complications in gestational-carrier pregnancies than in the same women's spontaneously conceived pregnancies. Although this design reduces confounding by stable maternal characteristics and excludes multiple births from the comparison, it does not isolate the contributions of IVF, oocyte source, embryo-transfer protocols, or other differences between pregnancies. Evidence concerning IVF more generally is mixed: Sabban et al. (19) reported increased obstetric morbidity compared with spontaneous conceptions, whereas Ganer Herman et al. (20), comparing singleton IVF and naturally conceived pregnancies in the same women and excluding oocyte donation, found no independent association between IVF and preterm birth, small-for-gestational-age birth, or pregnancy-induced hypertension after adjustment. Frozen–thawed embryo transfer has been associated with higher odds of hypertensive disorders of pregnancy and preeclampsia compared with spontaneous conception in singleton pregnancies (OR 1.74 and 1.82, respectively) (21).

Evidence from donor-oocyte pregnancies provides additional, although indirect, insight into the possible significance of genetic unrelatedness between the pregnant woman and the fetus. In a matched cohort of healthy women younger than 40 undergoing single embryo transfer, donor-oocyte pregnancies were associated with higher adjusted odds of hypertensive disorders, cesarean delivery, and postpartum hemorrhage than pregnancies using autologous oocytes (22). A meta-analysis of singleton pregnancies likewise identified associations between oocyte donation and several adverse outcomes, although differences in preterm birth and low birth weight were no longer statistically significant in analyses adjusting for preeclampsia (23). Supporting a possible immunological contribution, van Bentem et al. (24) found that greater maternal–fetal HLA class II mismatch was associated with preeclampsia within donor-oocyte pregnancies. These findings suggest that immunogenetic differences may contribute to obstetric risk, but they do not establish that genetic unrelatedness alone causes the observed complications or quantify its contribution specifically in gestational surrogacy. The risks women undertake remain ethically significant despite uncertainty about their precise causes; counselling, informed consent, and risk-reduction strategies should therefore address both the observed outcomes and the limitations of causal attribution.

Psychological concerns include possible emotional ambivalence, distress, and the evolving relationship between the carrier, intended parents, and child (25, 26). Religious traditions also contribute to divergent moral evaluations; Christian and Islamic objections have often emphasized marital integrity, lineage, and the commodification of reproduction (1) The most persistent controversy, however, concerns exploitation and commodification. Critics argue that pregnancy may be transformed into a contractual service and that economic inequality can undermine the voluntariness of consent, particularly in cross-border commercial arrangements. Proponents counter that denying women the capacity to choose compensated reproductive labor may itself be paternalistic and inconsistent with reproductive autonomy. Contractual attempts to regulate diet, travel, sexual behavior, abortion, or other aspects of pregnancy make this tension especially visible: intended parents have legitimate interests in the pregnancy, but those interests cannot convert the carrier's body into an object of contractual control.

## 6 Current trends in moral and ethical considerations toward gestational surrogacy

The literature published since 2015 reveals a field dominated by normative rather than empirical scholarship (Supplementary Table 1**).** Three broad developments are particularly evident. First, the debate has moved beyond the earlier opposition between autonomy and commodification toward more sophisticated accounts of structural vulnerability, relational autonomy, and the social conditions under which consent is given (8, 27–34). Second, the interests of the child have become increasingly prominent, particularly with respect to legal parentage, citizenship, knowledge of origins, and recognition of the gestational relationship (35–39). Third, although much of the academic and professional literature remains regulationist, rejecting both blanket prohibition and laissez-faire markets, recent political initiatives have moved the public discourse toward a more explicitly abolitionist vocabulary (16, 40–42).

The Casablanca Declaration for the Universal Abolition of Surrogacy, published on 3 March 2023 (43), represents the clearest contemporary expression of this abolitionist position. Although it is not legally binding, it proposes a normative basis for national legislation and an eventual international convention prohibiting surrogacy. The Declaration rejects distinctions between commercial and altruistic, traditional and gestational, and domestic and cross-border surrogacy, arguing that the practice itself is incompatible with human dignity because it instrumentalizes pregnancy, commodifies women and children, and exposes vulnerable women to exploitation. It therefore calls for prohibition of surrogacy agreements, agencies and intermediaries, sanctions against facilitators, extraterritorial measures against citizens commissioning surrogacy abroad, and ultimately universal abolition.

Directive (EU) 2024/1712, which amended the EU Anti-Trafficking Directive, expressly includes the exploitation of surrogacy among the forms of exploitation covered by the trafficking framework (94). Its application nevertheless requires fulfilment of the constitutive elements of trafficking, including the applicable means criterion. Recital 6 specifically identifies coercion or deception of women into acting as surrogate mothers and expressly preserves national rules on surrogacy, including criminal and family law. The Directive thus addresses trafficking for the exploitation of surrogacy; it neither equates surrogacy itself with trafficking nor establishes a general EU prohibition of surrogacy. (Directive (EU) 2024/1712, recital 6 and Article 1(1) (94)).

At the level of political discourse, the European Parliament's resolution of 13 November 2025 on the Gender Equality Strategy condemns surrogacy in terms of reproductive exploitation and the use of women's bodies for gain, with particular concern for especially vulnerable women in third countries. This non-binding resolution expresses a political position and does not itself create a legal prohibition. Its language can be interpreted as overlapping with elements of an abolitionist moral framework, but that overlap does not establish formal endorsement of the Casablanca Declaration or a commitment in binding EU law to universal abolition (44).

These distinctions leave open the central ethical question: is exploitation intrinsic to surrogacy, or does it arise from particular socioeconomic and regulatory conditions? Neither the Directive's targeted trafficking provisions nor the existence of political calls for abolition resolves that question. Its assessment is central to evaluating the ethical justification for prohibition or regulation, alongside evidence about the capacity of either approach to prevent harm and protect the rights of those involved.

## 7 The novelty of contemporary surrogacy abolitionism

Surrogacy abolitionism itself is not a new ideological position. Its central claims, that surrogacy commodifies women's reproductive capacities, facilitates exploitation, and reduces children to objects of exchange were already prominent within radical feminist critiques of reproductive technologies in the 1980s. These arguments were particularly associated with the Feminist International Network of Resistance to Reproductive and Genetic Engineering (FINRRAGE), which opposed surrogacy as part of a wider struggle against the commercial and patriarchal control of reproduction. In the United States, the Baby M controversy similarly prompted feminist scholars to advocate prohibition rather than regulation. Contemporary abolitionist discourse therefore draws substantially on an established repertoire of arguments concerning commodification, reproductive slavery, and the limits of contractual consent (45).

What is distinctive about the movement that emerged in the mid-2010s is its organizational form, geographical reach, and engagement with a globalized surrogacy market. Although abolitionist arguments had begun to regain public visibility slightly earlier, 2015 marked an important stage in their institutionalization. Stop Surrogacy Now was launched that year as an international campaign calling for a worldwide prohibition of surrogacy. In Europe, feminist and human-rights organizations increasingly coordinated their advocacy through international institutions, while Italian feminist mobilization for a universal ban became publicly visible in December 2015 (46, 47). These developments culminated in the establishment of the International Coalition for the Abolition of Surrogate Motherhood (ICASM) in 2018, which provided a formal transnational structure for abolitionist organizations (40).

This mobilization subsequently extended beyond Europe and North America. In Latin America, abolitionist organizations, including the regional Coalition Against Trafficking in Women, joined an international campaign against what they termed “reproductive exploitation” (48). The movement thus developed through the interaction of national feminist campaigns, anti-trafficking organizations, human-rights advocacy, and international coalitions. Its institutional targets also expanded to include bodies concerned with cross-border family law and international governance, such as the Council of Europe and the Hague Conference on Private International Law.

A further development occurred in 2025 within the United Nations human-rights system. Reem Alsalem, the Special Rapporteur on violence against women and girls, its causes and consequences, addressed surrogacy in her report to the General Assembly, The different manifestations of violence against women and girls in the context of surrogacy (49). An accompanying OHCHR press release in October 2025 publicized her call to recognize surrogacy as a system of violence, exploitation and abuse against women and to abolish it globally (50). This intervention illustrates the articulation of abolitionist arguments through an independent UN expert mandate, extending their visibility within international human-rights debate. The mandate's institutional role should nevertheless be distinguished from intergovernmental decision-making: the Special Rapporteur's analysis and recommendations do not themselves constitute binding international law or an adopted position of the United Nations as a whole (50).

The novelty of contemporary surrogacy abolitionism should therefore be understood as a process of resurgence and institutionalization rather than the invention of a new position. First, previously dispersed feminist critiques have been consolidated into named organizations and coordinated international campaigns. Second, abolitionist arguments developed during the 1980s have been redirected toward a commercial and transnational reproductive market involving fertility clinics, intermediaries, intended parents, and gestational workers across multiple jurisdictions. Third, the movement has assembled a broader and sometimes politically heterogeneous coalition in which radical or difference feminists interact with anti-trafficking organizations and, in some national contexts, religious and socially conservative actors (45, 51).

Some of the more innovative theoretical developments have emerged in response to this abolitionist resurgence. Scholars who conceptualize surrogacy as reproductive or gestational labour argue that exploitation should be addressed through labour rights, contractual safeguards, healthcare protections, and stronger regulation rather than prohibition (52–54). Contemporary abolitionism is therefore novel primarily as a coordinated transnational social movement confronting the globalization of commercial surrogacy, rather than as a new body of ideas.

The characterization offered here should nevertheless be read with some caution. Much of the historical interpretation rests on scholarly analyses of abolitionist campaigns, several of which adopt a critical position toward abolitionism. These perspectives may influence how the movement's historical continuity, political alliances, and claims to novelty are interpreted. Such analyses should therefore be read alongside primary statements and institutional documents, including Alsalem's 2025 (49) report and the accompanying OHCHR communication (50, 55). These sources provide direct evidence of the positions advanced by their authors, but their recommendations should be distinguished from empirical findings about the prevalence of harm, the comparative effectiveness of regulation, and the existence of an international consensus.

## 8 The fault lines: commodification, exploitation, and autonomy

Three normative frames organize much of the contemporary debate. The first is commodification. Abolitionist and restrictionist accounts argue that surrogacy, particularly when commercial, converts women's reproductive labor and potentially children into objects of exchange, thereby violating human dignity. Some versions of this argument extend to altruistic surrogacy because removing payment does not necessarily remove instrumentalization. Others contend that the morally objectionable features arise from abusive practices rather than from exchange itself and can therefore be addressed through targeted legal regulation (56).

The second frame is exploitation. Research on transnational surrogacy, particularly in India, has shifted attention from isolated transactions toward background inequality. Wilkinson (57), Sifris (58), and Fellowes (28), distinguish transactional exploitation from structural exploitation, emphasizing that formally voluntary agreements may still be shaped by poverty, gender inequality, restricted alternatives, and unequal bargaining power.

The third frame is reproductive autonomy. Permissive accounts regard the decision to become a gestational carrier as an exercise of bodily and reproductive self-determination and question whether prohibition itself unjustifiably restricts women's choices (58). Francis (59) similarly argues that commercial and altruistic surrogacy can be ethically acceptable when coercion and exploitation are adequately controlled. Bandelli (47) captures the resulting polarization: surrogacy is interpreted either as commodification and reproductive exploitation or as reproductive freedom, bodily autonomy, and potential empowerment. The persistence of these same moral frames across different jurisdictions suggests that the ethical vocabulary has become global even while law remains highly fragmented.

## 9 Commercial versus altruistic surrogacy

The distinction between commercial and altruistic surrogacy remains one of the principal organizing categories of law and professional ethics, but its moral clarity has eroded. Clinical and professional guidance generally regards non-commercial gestational surrogacy as more defensible when accompanied by informed consent, independent advice, protection from exploitation, and attention to the child's welfare (5, 60, 61). Under this model, reimbursement compensates reasonable pregnancy-related costs without converting gestation into a market transaction.

A substantial recent literature, however, questions whether commercial and altruistic arrangements can be cleanly separated. Lee (62) and Rudrappa (63) argue that exploitation or domination can arise in either model. Familial obligation, emotional indebtedness, and gendered expectations may constrain autonomy in altruistic arrangements just as economic inequality may constrain it in commercial ones. Ethnographic observations from India similarly suggest that eliminating payment can relocate rather than eliminate coercive relationships, particularly when surrogacy is restricted to relatives (64). Rudrappa and Collins (65) further show how narratives of altruism and compassion can coexist with commercial exchange and may obscure unequal power relations.

Reimbursement practices introduce an additional complication. In the United Kingdom and Canada, frameworks commonly described as altruistic permit pregnancy-related expenses to be reimbursed, although their legal rules differ. UK guidance acknowledges that reasonable expenses vary according to individual circumstances and may be estimated by the parties at the outset (66). Canadian rules permit reimbursement across several categories, including care of dependants, additional pregnancy-related food costs, and certain medically recommended services, potentially including household assistance (67). The breadth of these categories creates a boundary that requires careful interpretation and oversight. Health Canada expressly warns that reimbursement must not provide monetary gain or disguise payment for surrogacy. In the United Kingdom, the Nuffield Council on Bioethics (68) has similarly identified concerns that uncertainty over reasonable expenses creates scope for hidden commercialization. These concerns do not establish that legitimate reimbursement is equivalent to commercial remuneration: covering costs and losses remains conceptually distinct from paying for reproductive labour. They do, however, strengthen the case for examining what payments cover, how they are calculated, and whether they confer a net financial benefit, rather than relying on the altruistic label as evidence of ethical acceptability.

Conversely, the fair-labor argument challenges the assumption that non-payment is ethically superior. Blazier and Janssens (69) and Feiglin and Savulescu (70 argue that refusing payment for demanding reproductive labor may itself be exploitative and may restrict autonomy. From this perspective, ethically regulated compensation, combined with independent legal representation, insurance, counseling, and professional oversight, may better recognize the carrier's contribution than an altruistic-only regime. The contemporary debate has therefore moved away from the simple equation of commercial surrogacy with exploitation and altruistic surrogacy with ethical acceptability. The more consequential questions concern voluntariness, bargaining power, distribution of benefits and risks, and the institutional safeguards surrounding the arrangement.

## 10 Managing competing interests

Ethical analysis of gestational surrogacy should consider the interests of the gestational carrier, the intended parent(s), and the resulting child, together with broader societal concerns about justice and exploitation (71, 72). Professional ethical guidance emphasizes respect for the carrier's autonomy and dignity, protection of her physical and psychological health, voluntary and informed consent, and access to appropriate care and support during pregnancy and after birth (71, 73). Concerns about meaningful consent are not confined to the former commercial surrogacy market in India. Ethnographic research in Georgia has described difficulties understanding contractual rights and financial barriers to withdrawal, raising questions about the voluntariness and continuing nature of consent (74). More recently, investigative reporting from Georgia has documented migrant gestational carriers’ accounts of language barriers and inadequate explanations of medical treatment (75). Although these sources do not establish the prevalence of inadequate consent across commercial surrogacy arrangements, they illustrate why a signed contract cannot, by itself, demonstrate informed and voluntary consent to subsequent medical procedures.

The intended parents' interest in procreation is ethically significant but does not confer authority over the carrier's medical decisions. Contractual provisions addressing termination, fetal reduction, prenatal diagnosis, confidentiality, or life-sustaining treatment illustrate the limits of contract when bodily autonomy is at stake. Intended parents also retain moral responsibilities toward the child, including when prenatal abnormalities or disability complicate their original reproductive expectations (76).

Societal interests include reproductive justice, gender equality, and confidence in institutions governing assisted reproduction. Feminist analyses of surrogacy remain divided, particularly over the relationship between reproductive autonomy, commodification, and structural inequality (40). Autonomy-based arguments emphasize women's capacity to choose surrogacy and question whether prohibition unjustifiably restricts reproductive decision-making (58). Feminist critiques of commodification, by contrast, question whether commercial arrangements subject women's reproductive capacities and children to inappropriate market valuation (77). Relational-autonomy approaches complicate this opposition by assessing individual agency within the social and institutional conditions that shape available choices, drawing attention to vulnerabilities and structural exploitation that an exclusive focus on individual consent may overlook (31). Liberal approaches tend to view adequately protected surrogacy as compatible with autonomy, whereas radical-feminist and some religious or care-ethics approaches understand it as commodification within unequal social structures. Relational-autonomy frameworks attempt to bridge this divide by recognizing autonomous choice while insisting that structural vulnerability must be incorporated into assessments of whether consent is genuinely meaningful.

## 11 Disentangling the genetic, gestational, and legal relationship with the child

The ethics and regulation of surrogacy require a clear distinction between genetic connection, gestational connection, and legal parenthood. Genetic connection concerns the provision of the gametes from which a child develops; gestational connection arises through pregnancy and childbirth; and legal parenthood is the status through which a legal system identifies a child's parents and attaches rights and responsibilities to that relationship. These dimensions may coincide, but assisted reproduction can separate them. The moral significance of a genetic or gestational relationship therefore requires consideration independently of the question of what legal status should follow from it.

Accounts of parenthood offer different justifications for assigning parental rights and responsibilities. Kim (78) distinguishes genetic, gestational, causal, and intentional accounts, which respectively emphasize genetic relatedness, pregnancy, responsibility for bringing a child into existence, and the intention to become a parent. These accounts should be understood as competing or potentially complementary normative explanations, rather than interchangeable descriptions of legal parenthood. Legal parenthood exists whether the underlying relationships coincide or diverge. It should also be distinguished from the actual provision of care and from the allocation of custody or decision-making authority, which need not correspond completely to parental status.

Within this framework, acknowledging the significance of gestation does not, without further justification, establish that the woman who gives birth must be recognized as, or remain, the child's legal mother. Mulligan (38) argues that surrogacy law should take the gestational relationship seriously while recognizing that respect for that relationship need not require the attribution of maternal status. This distinction does not make gestation irrelevant. Rather, it requires separate consideration of how the surrogate's contribution and relationship with the child should be acknowledged and protected. Respect for gestation may have implications for information about origins, the treatment of the surrogate, and the continuing relationships among those involved, even where legal parenthood is assigned to the intended parents.

The ethical implications of gestation nevertheless remain contested. Arguments that pregnancy generates parental rights appeal, among other considerations, to gestational labour and the development of a relationship with the fetus. Porter (79) critically examines an account grounded in gestational labour and bonding, questioning whether it successfully establishes a right to parent the particular child. The existence of this disagreement cautions against treating either gestation or reproductive intention as a self-evidently sufficient basis for legal parenthood. Similarly, a woman's agreement to undertake a pregnancy for others is relevant to the allocation of parental status, but the legal implications of that agreement require attention to informed consent, possible changes in circumstances, and safeguards against exploitation.

The approach defended here is therefore pluralist. It gives substantial weight to the parties’ reproductive intentions without treating their agreement as sufficient to resolve every question concerning the child's status. A defensible framework should consider the significance of gestation, the surrogate's autonomy, the intended parents' undertaking to assume responsibility, and the child's independent interests. It should also distinguish consent to medical treatment and pregnancy from consent concerning legal parenthood. Rules addressing the timing and possible withdrawal of consent require explicit justification, rather than an assumption that pre-conception agreement conclusively determines subsequent parental status.

A child-centred analysis must further distinguish access to information about origins from the allocation of legal parenthood. Preserving information about genetic contributors and the woman who gave birth can protect an important dimension of the child's identity without necessarily conferring legal parental status on each person concerned. Mulligan (38) emphasizes the importance of transparency about gestational origins, while Park-Morton (37) argues that relationships with non-genetic intended parents also contribute to the identity of children born through surrogacy. These perspectives support an understanding of identity that encompasses both origins and lived family relationships.

Disclosure and record preservation should therefore be treated as important safeguards, rather than as an exhaustive response to the child's identity interests. Article 7 of the Convention on the Rights of the Child addresses birth registration, nationality, and, as far as possible, knowledge of and care by parents. Article 8 protects identity, including nationality, name, and family relations. These provisions provide a framework for evaluating surrogacy arrangements, but do not themselves specify how genetic, gestational, and intended parenthood should be prioritized. The child's best interests and developing views also require consideration under Articles 3 and 12 (92).

Legal certainty and nationality raise related but distinct questions. A clear domestic parentage rule does not necessarily ensure recognition in another jurisdiction or establish the child's nationality. Protection therefore requires attention to the interaction between parentage, registration, nationality, and cross-border recognition. The Law Commission and Scottish Law Commission (80), for example, address intended parenthood, access to origin information, and nationality safeguards as separate elements of their proposed reforms. Their proposed domestic pathway would recognize intended parents from birth, subject to screening, independent advice, counselling, and provisions concerning the surrogate's withdrawal of consent. This illustrates a possible regulatory approach; it does not establish that intention alone determines parenthood or that recognition from birth is the only defensible model.

The distinction between conceptual analysis and existing law must also remain explicit. In England and Wales, the surrogate is the legal mother at birth, while a parental order subsequently changes legal parentage. Depending on the circumstances, an intended father may already be the legal father at birth (81). Existing law thus already distinguishes genetic connection, gestation, and legal status, although the weight assigned to each remains open to normative evaluation.

The conclusion is that gestation should neither be disregarded nor assumed, without further argument, to determine legal motherhood. Equally, recognition of the intended parents' role should not displace scrutiny of consent or the child's independent interests. The task is to justify an allocation of parental status that provides stable and enforceable responsibilities while protecting the surrogate's autonomy, preserving meaningful access to origins, and avoiding uncertainty concerning the child's family relationships and nationality. Distinguishing these questions strengthens the analysis without presupposing that children's rights necessarily require a single model of legal parenthood.

## 12 Cultural, national, and regulatory variation

National approaches to surrogacy have diverged rather than converged (1). India illustrates a transition from a major destination for transnational commercial surrogacy to a highly restrictive altruistic regime under the Surrogacy (Regulation) Act, 2021. Critics argue that the new framework excludes single and LGBTQ + intended parents, relies heavily on family-based altruism, and may displace rather than eliminate exploitation. Ukraine and Russia historically developed comparatively permissive commercial systems, although both have subsequently faced political, legal, and, in Ukraine, wartime disruption. The United States remains a state-level patchwork, with jurisdictions ranging from regulated commercial surrogacy to prohibition and no comprehensive federal framework.

The United Kingdom, Canada, Australia, Greece, and the Netherlands exemplify varying forms of altruistic or non-commercial regulation, although restrictions on compensation may contribute to shortages of carriers and cross-border reproductive travel. By contrast, France, Germany, Italy, Spain, Austria, and several other continental European jurisdictions prohibit surrogacy, with Italy extending criminal liability to citizens who pursue surrogacy abroad. Israel represents a distinct highly regulated model in which surrogacy is permitted subject to state oversight and approval, and has been described as a regulationist model (1).

Religious and cultural traditions further shape these regulatory differences. Catholic teaching and many Islamic approaches are restrictive, particularly because of concerns about commodification, marital integrity, and lineage. Other traditions are more heterogeneous: Buddhist analyses may focus on motivation rather than categorical prohibition, while Jewish and Hindu perspectives encompass multiple positions. Such variation cautions against assuming that a single ethical vocabulary will automatically produce international legal convergence (1).

## 13 Cross-border surrogacy: the unresolved challenge

Cross-border surrogacy exposes the limitations of purely national regulation. Restrictions on domestic access may lead intended parents to seek arrangements in jurisdictions where surrogacy is permitted or where oversight is less developed (16). In such settings, socioeconomic inequalities and intermediary practices may compound unequal bargaining power and risks of exploitation (16, 69). Differences between national rules governing legal parentage and nationality can also leave children with uncertain or inconsistently recognized legal status across borders (82). National restrictions may therefore displace some arrangements abroad without necessarily resolving the vulnerabilities they seek to address (16).

For this reason, much of the international literature converges on the need for greater coordination, even where authors disagree about whether the ultimate objective should be prohibition or regulated access. The Hague Conference on Private International Law's work on parentage is frequently identified as a plausible institutional route toward greater legal certainty. Yet international harmonization presents its own ethical difficulty: a framework designed to prevent exploitation may inadvertently legitimize practices considered unacceptable in some societies, while a prohibitionist convention may impose one contested moral position across culturally and legally diverse jurisdictions. Cross-border surrogacy therefore remains the setting in which the tension between reproductive freedom, protection from exploitation, and international legal pluralism is most difficult to resolve.

## 14 A middle path: regulated gestational surrogacy

Israel regulates domestic gestational surrogacy through the Embryo Carrying Agreements (Agreement Approval and Status of the Newborn) Law, enacted in 1996. Rather than leaving the arrangement primarily to private contract, the law requires every surrogacy agreement to receive prior authorization from a state-appointed Approval Committee. The seven-member committee comprises two specialists in obstetrics and gynecology, a specialist in internal medicine, a clinical psychologist, a social worker, a jurist, and a representative of the religion of the parties. Since January 2022, the domestic procedure has been available to eligible couples, including same-sex couples, and to single women and men, provided that at least one intended parent has a genetic relationship with the resulting child (Israeli Ministry of).

The committee reviews the proposed agreement and the medical and psychological suitability of the intended parents and gestational carrier. It must be satisfied that the parties have entered the agreement voluntarily, understand its meaning and consequences, and meet the statutory eligibility and health requirements. The gestational carrier must be an Israeli resident, have previously experienced pregnancy and childbirth, and meet specified age, health, and reproductive-history criteria. The committee also reviews payments and may authorize reimbursement of expenses and reasonable compensation, including compensation for loss of income, time, and temporary loss of earning capacity. Payments or benefits outside the approved agreement are prohibited. This system is intended to place medical, psychological, legal, and financial safeguards around the arrangement before treatment begins, while providing a predetermined legal process for recognizing the intended parents and protecting the status of the resulting child (Israeli Ministry of).

The Israeli framework should be assessed alongside other systems permitting compensated surrogacy under substantial regulatory safeguards, particularly New York's Child-Parent Security Act. New York requires independent legal representation, medical evaluation and informed consent, financial safeguards, and insurance protection, while regulating surrogacy intermediaries. It also expressly protects the gestational carrier's authority over medical decisions, including pregnancy continuation or termination, and provides for intended parentage at birth under compliant agreements (83, 92–94, 98).

Recent law-reform initiatives in the United Kingdom and Australia provide further points of comparison. In 2023, the Law Commission of England and Wales and the Scottish Law Commission proposed a new pathway for domestic surrogacy under which intended parents could acquire legal parenthood from birth, subject to safeguards and the gestational carrier's right to withdraw consent. The proposed pathway includes screening, independent legal advice, counselling, and oversight by regulated non-profit surrogacy organisations. It retains an altruistic framework and clarifies permitted payments. These recommendations should be distinguished from enacted law: in April 2025, the UK Government stated that it was unable to prioritise their implementation (84).‏

In Australia, the Australian Law Reform Commission's Review of Surrogacy Laws examines options for greater consistency across jurisdictions, improved protection of participants, recognition of parentage following overseas arrangements, and children's access to information about their origins. Its 2025 Discussion Paper proposes a stronger regulatory framework, including a preferred option of national oversight, licensing of surrogacy support organisations, and clearer rules governing financial arrangements (85). These proposals illustrate an approach centred on improving the organisation and accountability of surrogacy, although their protective effects cannot be assumed in advance of implementation and evaluation.‏

These initiatives share with the Israeli framework an emphasis on safeguards established before treatment and clearer institutional responsibility. They nevertheless differ in their approaches to compensation, oversight, and legal parentage. Their relevance is therefore comparative: they demonstrate different ways of structuring regulated surrogacy, while leaving open the empirical question of which arrangements most effectively protect participants.

Israel's distinctive contribution is mandatory approval of each agreement by a national multidisciplinary committee before treatment begins. This adds an institutional opportunity to scrutinize consent, medical and psychological assessments, contractual terms, and compensation beyond the parties’ professional advice (86, 87, 95–97). Its potential advantage is therefore the integration of safeguards within individualized public review. Nevertheless, greater administrative oversight does not itself demonstrate stronger protection: it may also introduce delay, restrict access, and expand discretionary judgments about reproductive eligibility. Furthermore, New York's explicit protection of medical autonomy and recognition of parentage at birth are substantial strengths. The Israeli model is therefore better characterized as one potentially defensible compromise between reproductive autonomy and protective oversight, rather than as a demonstrably superior regulatory model. Establishing comparative superiority would require evidence concerning consent quality, carrier welfare, access, and outcomes for children.

The available literature provides some support for the protective rationale of this model, but it does not establish that Israeli regulation causes a measurable reduction in harm. The evidence cited here does not establish that the Israeli approval system causes a reduction in exploitation or produces superior outcomes compared with alternative regulatory models. Teman and Berend's (88) comparative ethnographic research found that Israeli gestational carriers generally supported governmental regulation and often sought greater state responsibility. These findings illuminate participants’ perceptions of oversight, but they do not measure its causal effects on exploitation, health, or long-term welfare. Although these findings do not demonstrate a lower incidence of harm, they suggest that the Israeli participants studied generally regarded state oversight as a source of protection rather than as an inherently coercive intervention.

Evidence from other regulated settings similarly indicates that surrogacy is not necessarily associated with adverse outcomes and that positive outcomes are more likely when arrangements take place openly within systems providing independent advice, informed consent, healthcare, legal protection, and institutional accountability (42). These findings support the rationale for robust regulation, but they cannot isolate the effect of the Israeli committee or establish the superiority of every aspect of the Israeli model. Regulation may itself create burdens, exclude some prospective participants, or provide formal protections that are not implemented consistently in practice. It also cannot eliminate all inequalities between gestational carriers and intended parents or resolve objections that regard commodification and the planned relinquishment of a child as intrinsic to surrogacy.

A further limitation concerns the territorial reach of the Israeli framework. Its principal safeguards apply to arrangements undertaken within the domestic system. They do not provide equivalent protection to gestational carriers involved in arrangements entered into by Israeli intended parents abroad. Zafran and Hacker (89) found that states may have limited capacity and motivation to protect gestational carriers when surrogacy takes place in another jurisdiction. Restrictive access to domestic surrogacy may therefore contribute to cross-border arrangements in which oversight is weaker and responsibility for safeguarding the gestational carrier is fragmented among states, clinics, agencies, and intended parents.

The Israeli model should consequently be understood as an example of a regulatory system designed to reduce identifiable risks, rather than as proof that state authorization totally eliminates exploitation or guarantees better outcomes. Its principal contribution is to demonstrate how surrogacy can be brought within a public framework of prospective review, professional assessment, controlled compensation, and legal accountability. The qualitative evidence from Israel and the broader evidence from regulated jurisdictions are consistent with the proposition that such safeguards may reduce opportunities for harm. Whether the Israeli system achieves lower rates of exploitation or adverse outcomes than alternative regulatory or prohibitionist models remains an important question requiring direct comparative and longitudinal research.

Where donated gametes are used, gamete donors constitute an additional group of stakeholders whose interests cannot be subsumed under those of intended parents or gestational carriers. Their moral claims include informed and voluntary consent, protection of health and privacy, and adequate information about the potential uses of their gametes and the implications of future genetic identification or contact. Egg donors require particular attention because they undergo ovarian stimulation and oocyte retrieval (90). Israel's domestic egg-donation framework provides safeguards including consent review, limits on repeated donation, regulated compensation, and a right to withdraw consent to reproductive donation before fertilization (Israel Ministry of). These protections arise from the egg-donation framework and should not be inferred from approval of a surrogacy agreement alone. Moreover, their application to donors abroad cannot be assumed: a 2025 parliamentary report identified limitations in Israeli oversight of foreign clinics supplying donated eggs (91). Any ethical assessment of the Israeli model must therefore consider the circumstances of gamete procurement alongside the conditions of gestation. Recognition of donors’ independent interests does not itself entail legal parenthood, but it does require their inclusion in the analysis.

Thus, contemporary approaches to gestational surrogacy can be situated along a continuum, ranging from permissive systems with limited state oversight to abolitionist frameworks that seek to prohibit the practice altogether. The purpose of this review is not to determine categorically which of these approaches is superior, but to examine the concerns identified in the literature and the implications of the different policy responses proposed.

Permissive approaches are commonly grounded in reproductive autonomy and in recognition of the needs of individuals and couples for whom gestational surrogacy may represent the only realistic pathway to parenthood. When effective safeguards are absent, however, socioeconomic inequality and unequal bargaining power may expose gestational carriers to exploitation, inadequate informed consent, commodification of reproductive labor, excessive commercial intermediation, and, in extreme cases, coercive or trafficking-like practices. These concerns are central to the abolitionist position, which regards such harms as inherent, or sufficiently integral, to surrogacy to justify its prohibition. Abolitionist scholarship therefore makes an important contribution by drawing attention to the structural inequalities within which many surrogacy arrangements occur and by questioning whether consent alone can legitimize practices shaped by economic vulnerability.

Prohibition, however, also has significant ethical and practical implications. It removes an important, and, for some people, the only pathway to parenthood, including for women without a functional uterus, women for whom pregnancy would entail an unacceptable medical risk, male same-sex couples, and others who require the assistance of a gestational carrier to pursue genetically related parenthood. Adoption and other reproductive treatments cannot necessarily provide an accessible, suitable, or equivalent alternative in every case. Moreover, national prohibition does not necessarily eliminate either the demand for or the practice of surrogacy. Where arrangements remain available in other jurisdictions, prohibition may displace surrogacy across national borders and restrict access to those with sufficient financial resources. It may also direct intended parents toward jurisdictions in which medical, legal, and economic protections for gestational carriers and resulting children are less comprehensive. This possibility raises concerns about inequitable access, reproductive travel, the legal status and welfare of children, and the capacity to monitor and prevent exploitation.

The distinction emerging from the literature is therefore between harms that are necessarily inherent to surrogacy and harms that are produced or intensified by the conditions in which it takes place. The available evidence does not establish that regulation can eliminate every ethical concern associated with surrogacy, and poorly designed regulation may legitimize exploitative practices without adequately protecting gestational carriers. Nevertheless, where the identified harms arise principally from unequal bargaining power, inadequate healthcare, insufficient informed consent, unregulated intermediaries, or weak legal protections, the literature provides grounds for considering whether enforceable regulation may offer a more proportionate response than categorical prohibition.

One such approach is the Israeli model in which, the state recognizes surrogacy as a form of assisted family building while accepting responsibility for establishing and enforcing protections for everyone involved. An adequate regulatory framework should provide for the gestational carrier's independent and continuing informed consent, appropriate medical and psychological assessment, independent legal representation, protection of bodily autonomy throughout pregnancy, transparent and proportionate compensation, oversight of agencies and other intermediaries, clear procedures for establishing parentage, and protection of the rights and welfare of the resulting child. Regulatory systems should also be monitored continuously to determine whether these safeguards operate effectively in practice and whether particular groups remain disproportionately exposed to risk.

Accordingly, the conclusion drawn from the reviewed literature is not that regulation renders surrogacy ethically unproblematic or that reproductive aspirations should override the rights of gestational carriers. Rather, it is that the ethical assessment of surrogacy must consider both the documented risks of exploitation and the consequences of removing a reproductive service for which some individuals have no equivalent alternative. From this perspective, the central policy question is whether a regulatory environment can protect reproductive autonomy while substantially reducing exploitation, inequality, and commodification. The ethical legitimacy of a surrogacy arrangement may therefore depend less on its formal classification as “commercial” or “altruistic” than on the circumstances in which consent is obtained, risks and benefits are distributed, and the rights and dignity of the gestational carrier, intended parents, and resulting child are protected.

## 15 Conclusions

Over the past decade, the moral debate surrounding gestational surrogacy has become both more sophisticated and more polarized. The earlier shorthand in which commercial surrogacy was treated as inherently problematic and altruistic surrogacy as presumptively ethical has not survived sustained scrutiny. Contemporary scholarship increasingly recognizes that exploitation can arise through markets, families, agencies, structural poverty, gender inequality, or unequal bargaining power, while payment may in some circumstances represent recognition of reproductive labor rather than its commodification. At the same time, the child's interests have become more prominent, and secure parentage, citizenship, knowledge of origins, and protection from avoidable legal uncertainty are now central elements of responsible regulation.

The field nevertheless stands at a moral crossroads. One trajectory, exemplified most clearly by the Casablanca Declaration and echoed in increasingly restrictive European political discourse, treats surrogacy itself as incompatible with human dignity and seeks abolition. The other, reflected in much of the reproductive-medicine and bioethics literature, regards the principal harms as contingent rather than intrinsic and therefore favors carefully designed regulation. The decisive ethical question is consequently not simply whether surrogacy should be commercial or altruistic, but whether the practice can be organized in a manner that preserves meaningful consent, protects gestational carriers from exploitation, recognizes the interests of children, and avoids transforming reproductive vulnerability into a market opportunity.

No national framework can fully address these concerns while surrogacy remains transnational. A defensible future approach will require clearer minimum standards for carrier autonomy and health, independent counseling and legal representation, transparent and fair financial arrangements, secure parentage and citizenship for children, responsible oversight of intermediaries, and mechanisms for international cooperation. Whether such safeguards can reconcile the competing moral positions remains uncertain. What is increasingly clear, however, is that the ethical future of gestational surrogacy will be determined less by the label attached to an arrangement, commercial or altruistic, than by the distribution of power, risk, responsibility, and protection among all those involved.
