# Supplementary material for: Gestational surrogacy at a moral crossroads: autonomy, exploitation, and abolitionism
Source: Front Glob Womens Health. 2026 Oct 29;7:1973804. doi: 10.3389/fgwh.2026.1973804 (PMC13755830; doi:10.3389/fgwh.2026.1973804)
Supplement: Supplementary file 1 [file Datasheet1.pdf]

Gestational Surrogacy Ethics Corpus — Provenance, Reconciliation & Re-drawn PRISMA

Audit of the search-and-screen pipeline behind the evidence table, RIS library, and PRISMA flow delivered earlier in this session. All figures below were reconstructed from the retained pipeline state (the eight search result sets, the screening decisions, the full-text-retrieval flags, and the evidence-table export); no numbers are estimated.

---

1. The eight searches: queries, filters, limits, coverage, exclusions

Database coverage — one source only. Every record in this corpus came from a single academic index (Elicit's paper search, which draws primarily on the Semantic Scholar graph). **No other database was searched** — not PubMed, Scopus, Web of Science, Embase, PhilPapers, Westlaw, or any grey-literature source. Web search and clinical-trial search were available but were **not** used for this corpus. Every record carries a Semantic Scholar identifier; 318 of 400 also carry a DOI.

Execution window. The searches were run programmatically inside this session (September 2026). The pipeline did not log per-query wall-clock timestamps, so exact execution times cannot be reconstructed; the queries, filters, and limits below are recovered verbatim from the retained notebook and are exact.

Common parameters for all eight searches: date filter `year > 2014` (lower bound only — **no upper bound**, which is why four 2026-dated records were captured); retrieval limit **50 records per query**; ranking = the index's default relevance ranking; language = **unrestricted**.

#	Exact query string	Filter	Limit
1	`ethics of gestational surrogacy bioethics`	`+year:>2014`	50
2	`commercial surrogacy exploitation moral arguments`	`+year:>2014`	50
3	`altruistic versus commercial surrogacy ethics`	`+year:>2014`	50
4	`transnational cross-border surrogacy ethics regulation`	`+year:>2014`	50
5	`surrogate mother autonomy commodification feminist ethics`	`+year:>2014`	50
6	`surrogacy regulation India Ukraine United States comparative ethics`	`+year:>2014`	50
7	`gestational surrogacy informed consent rights of the child`	`+year:>2014`	50

8	surrogacy ethical issues review	+year:>2014	50

Coverage and exclusion accounting. 8 queries × 50 = **400 returned hits**, and the retained result set contains exactly 400 records — meaning **each query returned its full 50 and the results were concatenated with no cross-query de-duplication**. The only exclusion step was the abstract-level relevance screen (Section 4), which dropped **64** records and retained **336**. There were no other exclusion filters (no date-of-full-text, no journal-quality, no language exclusions). The 64 exclusions were made by an automated relevance classifier; per-record exclusion reasons were retained as screening rationales but collapse to a single ground: "no substantive engagement with the ethics/morality of gestational surrogacy."

---

2. Record-to-publication crosswalk and the 400 → 336 → 168 reconciliation

The accompanying crosswalk file (`surrogacy-record-crosswalk.csv`, 400 rows) assigns every search hit two stable IDs — a **record ID** (the per-hit citation handle used in the source files and RIS) and a **publication ID** (`PUB0001`…, shared by records that are the same underlying publication) — and flags, for each record, whether it was retained, whether full text was retrieved, and whether it fed the evidence table. De-duplication is by exact match on Semantic Scholar ID, DOI (case-normalised), or normalised title, merged transitively.

The three headline counts are **nested subsets of one pipeline, not separate searches**. The 168-row workbook is a **selected subset** of the 336 retained records, which are themselves a screened subset of the 400 hits. It is not a partial export and not a separate search.

Stage	Records (hits)	Unique publications	Duplicate records
Identified via database searching	**400**	293	107
Retained after abstract screening	**336**	241	95
— Full text retrieved	116	—	—
— Abstract-only	220	—	—
Exported to evidence table	**168**	127	41

Key reconciliation facts:

- **The 400 is a count of search hits, not distinct papers**. Because no de-duplication step was ever run, 107 of the 400 records are duplicates of another record in the set; the 400 hits represent **293 distinct publications**.
- **The duplication propagates downstream**. The 336 retained records are **241 distinct publications** (95 duplicate records survived screening because each duplicate was screened independently). The 168 exported rows are **127 distinct publications**.
- **168 = the substantively-extractable subset of 336**. A record was exported to the evidence table only if automated extraction returned non-empty content on at least one analytic dimension. The other 168 retained records returned "not mentioned" across all dimensions (overwhelmingly the abstract-only records) and were left out rather than filled with empty rows.
- **The RIS library holds all 336 retained records** (including the 95 duplicate records), so it and the 168-row workbook are consistent views of the same pipeline at two different depths.

---

3. Duplicate-group validation and the four 2026 entries

Candidate duplicate groups. Within the 168 exported rows, deterministic matching on DOI, Semantic Scholar ID, and normalised title yields **127 unique-publication clusters**, of which **27 contain more than one record** (accounting

for the 41 duplicate rows). This 127 is the same quantity as an external de-duplication pass that reported ~129 candidate groups; the small difference (127 vs 129) is expected — reference-manager de-duplicators use fuzzy author/title similarity that can split or over-merge a handful of clusters relative to exact key-matching. Either way, the operative finding is the same: **the workbook contains on the order of 40 duplicate rows that should be collapsed before the table is treated as one-row-per-study**. The crosswalk's `publication_id` / `is_duplicate` / `duplicate_of` columns identify exactly which rows to merge.

The four 2026-dated entries — resolved. All four are **distinct, real publications** (none are duplicates of each other or of earlier records). Two are genuinely 2026; two are metadata-mis-dated 2025 works that belong inside the stated 2015–2025 window:

Title (abbrev.)	Metadata year	DOI	True year	Verdict
Paid Surrogacy as a Relational Autonomy Reproductive Labour (Lithuania)	2026	`10.15388/stepp.2026.62.7`	**2026**	Genuine 2026; outside stated decade but captured by the open-ended `year>2014` filter
Understanding Surrogacy in the Light of Ethical Values: A Review (Indus J. Bioscience Res.)	2026	`10.70749/ijbr.v4i12.1657`	**2026**	Genuine 2026; same
Nunca un niño puede ser tratado como un encargo… (Argentina)	2026	`10.37767/2362-5325(2025)014`	**2025**	Mis-dated; DOI shows 2025
Сурогатне материнство в Україні та світі… (Ukraine)	2026	`10.33244/2617-4154-3(20)-2025-180-188`	**2025**	Mis-dated; DOI shows 2025

Recommendation: correct the two mis-dated years to 2025, and either retain the two genuine 2026 papers with the corpus title updated to ”2015–2026” or drop them to hold the stated decade. Neither choice changes any substantive finding of the synthesis.

---

4. Who screened and extracted; languages; retrieval; final selection

This was a **single-pass automated pipeline**, and it should be described as such rather than as a dual-reviewer systematic review:

- **Screeners / extractors**. There were **no human reviewers and no independent double-screening**. Title/abstract relevance screening was performed by one automated relevance classifier against a single inclusion instruction, with an inclusion threshold of **55 / 100** confidence. Data extraction was performed by one automated language-model pass applying a fixed five-field schema (main thesis; commercial vs. altruistic; competing interests; cultural/national context; normative stance), each field required to quote the source. No second reviewer adjudicated either stage; there is therefore **no inter-rater reliability** to report.
- **Languages assessed**. No language restriction was applied at any stage. The retained corpus is genuinely multilingual — English, Spanish, French, Italian, Ukrainian, and Lithuanian records are all present — and non-English records were screened and extracted in their original language (which is why some evidence-table cells are in Spanish, French, or Italian).

- **Full-text retrieval**. Full text was sought for all 336 retained records by automated best-effort fetching. It succeeded for **116** and failed for **220** (paywalls, no open-access copy, or unparseable pages). Retrieval was not retried and no manual document sourcing was performed.
- **Final selection**. Inclusion in the evidence table was decided **mechanically**, not by expert judgment: a record was exported if and only if automated extraction produced substantive content. This favours full-text records and systematically under-represents the 220 abstract-only records.

On reconstruction vs. a new search. The original methods were **fully recoverable** from retained state — the exact queries, filters, limits, screening threshold, extraction schema, and per-record retrieval flags are all documented above. A fresh revision-stage search was therefore **not required** and was not performed; running one would create a *different* corpus rather than document the existing one. If the goal shifts from auditing this corpus to upgrading it to systematic-review standard, the appropriate next step is a protocol-driven multi-database re-run (adding PubMed/PhilPapers/legal databases, a de-duplication stage, dual human screening, and manual full-text sourcing) — which would be a new project, not a reconstruction.

---

5. Redrawn flow (see accompanying figure)

The redrawn diagram corrects three things the first PRISMA flow blurred:

1. **It shows the missing de-duplication step**. 400 hits are marked as containing 107 duplicate records (293 distinct publications), making explicit that no de-duplication was run in-pipeline.
2. **It separates abstract-level mapping from full-text appraisal**. The 336 retained records split into a **full-text-appraised stream (116, close-read)** and an **abstract-only mapped stream (220)**. The 220 abstract-only records are labelled as *screened and mapped at abstract level only — not fully appraised studies.*
3. **It places the 168-row / 127-publication evidence table as a downstream extraction subset**, not as a separate search or a full-appraisal set.

Bottom line: the corpus is sound as an abstract-level *map* of a decade of surrogacy-ethics scholarship, but it is **not** a de-duplicated, dual-reviewed, fully-appraised systematic review, and the 220 abstract-only records must not be presented as appraised studies. The crosswalk and redrawn flow make the true structure auditable.
